# Analysis of the relationship between condylar changes and anterior disc displacement with reduction: a preliminary study

**DOI:** 10.1007/s11282-022-00617-y

**Published:** 2022-05-13

**Authors:** Hadeer Nasser Mohamed, Mostafa S. Ashmawy, Mena El-Erian Youssef Ekladious, Mary Medhat Farid

**Affiliations:** 1 Faculty of Dentistry, Department of Oral and Maxillofacial Radiology, Ain Shams University, Monazamet El Wehda El Afriqeya St., Cairo, Egypt; 2 Faculty of Medicine, Department of Radiodiagnosis, Ain Shams University, Cairo, Egypt

**Keywords:** Temporomandibular joint, Cone beam computed tomography, Magnetic resonance imaging, Disc displacement

## Abstract

**Objectives:**

To predict temporomandibular joint (TMJ) anterior disc displacement with reduction (ADDWR) from condylar shape, position, and dimensions obtained from CBCT images.

**Methods:**

This cross-sectional study was performed on 17 patients suffering from temporomandibular disorders diagnosed by history taking according to the chart of the American Association of orthodontists, clinical examination according to the Helkimo index and MRI. CBCT and MRI examinations were performed within one-week interval. Disc position, diagnosed by MRI was used as the gold standard. TMJs with posterior disc displacement or anterior disc displacement without reduction were excluded. Qualitative and quantitative analyses were performed on CBCT images to find the correlation between condylar variables and ADDWR. A logistic regression model was created to estimate ADDWR from condylar dimensions (height, width and depth).

**Results:**

Condylar shape and condylar position in the glenoid fossa were significantly correlated with ADDWR (*P* < 0.05). Condylar width, height and depth were significantly smaller in condyles with ADDWR compared to condylar dimensions in normal disc position. Logistic regression analysis could be used to predict the probability of anterior disc displacement with reduction from condylar dimensions.

**Conclusion:**

Condylar shape, position, and dimensions assessed by CBCT are significantly correlated with ADDWR of the TMJ. Substituting the values of condylar width, height and depth in the equation suggests the probability of ADDWR.

## Introduction

Internal derangement is the most frequent cause of temporomandibular joint disorders (TMD). It is described as an abnormal positional and functional relationship between the disc and articulating surfaces [1]. Anterior disc displacement with reduction (ADDWR) is the prevalent intra-articular TMD representing 41% of TMD diagnoses [2]. Additionally, ADDWR is discovered in up to 33% of asymptomatic individuals [3]. ADDWR is characterized by the progressive displacement of the articular disc against the mandibular condyle, accompanied by pain, clicking sounds as the disk resumes its normal position on jaw opening and in some cases limitation of mandibular movement [4].

Several studies suggested that changes in the condylar shape, condylar position and condylar dimensions are associated with ADDWR. It was reported that the normal condylar head has a convex configuration throughout [5] and any changes in this configuration may be attributed to a change in the disc position. And that there is a higher prevalence of ADDWR in angled condyles [6]. Condyles in patients with ADDWR generally showed a more posterior position and an increased size of anterior joint space [7, 8]. Also, condylar dimensions may be significantly associated with disc displacements of the TMJ [9].

MRI is the gold standard for TMJ disc examination [10]. As MRI is a non-invasive technique, free of ionizing radiation with superior soft-tissue resolution. Nowadays, CBCT is widely used in the majority of dental fields, being a 3D imaging modality with lower dose, lower cost, high spatial resolution and smaller foot print [11]. Therefore, this research was carried out to test the ability of quantitative and qualitative CBCT assessment of the osseous components of the TMJ in predicting ADDWR. In addition, logistic regression derived from this study may lead to the use of computer aided CBCT assessment in the diagnosis of ADDWR.

## Patients and methods

This cross-sectional study was approved by the research ethics committee of our institute with the approval number FDASU-RECim121816. A power analysis was designed to have adequate power to apply a statistical test of the null hypothesis that there is no difference between tested techniques. According to the results of Schnabel et al. [12] and by adopting an alpha of 0.05 (5%) and a beta of 0.10 (10%) i.e. power = 90% the predicted sample size (*n*) was found to be (26) TMJs i.e. 13 patients. Because we planned to exclude ADDWOR and PDD, we anticipated a dropout rate of 30%. The adjusted sample size was (34) TMJs i.e. 17 patients. Sample size calculation was performed using G*Power version 3.1.9.7 [13].

### Patients’ selection

Seventeen patients suffering from TMD were included in the current study. They were informed about the aim, steps, benefits and risks of the study and signed an informed consent. They were recruited from the specialized TMD clinic affiliated to the institute.

Patients ranging in age from 18 to 55 years having TMD diagnosed by a combination of history and clinical examination. History of the patient’s complaints were taken according to the chart of American Association of Orthodontists [14] then clinical examination was performed according the Helkimo index; scores ranged from 0 to 20 [15] (Table 1). This index evaluates the functional capacity of the masticatory system. It classifies individuals according to four signs: (1) function impairment, (2) TMJ pain during palpation, (3) impaired range of mandibular movement, and (4) muscle tenderness. If the sum of the scores was zero this indicates normal TMJ, if the sum of scores was from one to four this indicates a moderate TMD while if the sum of the scores was from five to twenty this indicates a severe TMD. In the present study, we included patients with scores ranging from five to twenty (severe TMD), not responding to conservative treatment and were planned to be treated by arthrocentesis. Two image acquisitions were performed for each patient included in our study within one-week interval. MRI to detect disc position and CBCT to rule out any bone pathology. Patients with TMJ fractures, cysts, tumors, inflammatory or systemic diseases affecting the TMJ like rheumatoid arthritis were excluded.

**Table 1:** The Helkimo index modified by Athanasiou and Melsen [15]

Criteria	Score = 0	Score = 1	Score = 5
1.Mandibular mobility	Normal	Reduced	Severely reduced
2. TMJ function	Plane movement without sounds and deviations	Sounds in one or both joints and/or deviations	Locking or luxation
3. Muscular pain on palpation	No pain	Pain at 1–3 sites	Pain at 4 > sites
4. TMJ pain on palpation	No pain	Lateral pain	Distal pain

### Imaging methods

Bilateral MRI scans of the TMJs were performed for each patient using a Philips Ingenia 1.5 T closed MRI unit (Philips Healthcare, Best, Netherlands). The patients were asked to lay down in a supine position, with the Frankfort plane parallel to the scanner gantry, and the sagittal plane perpendicular to the floor. Bilateral TMJ surface coils were used for optimal imaging of the TMJ, with a small field of view in order to achieve a higher signal-to-noise ratio.

Sequential axial, sagittal and coronal cuts of the right and left sides were obtained both in the closed mouth (maximum intercuspation) and maximum opening positions. Fast spin echo sequence was used to obtain three mm thick images T1, T2 and PD images. T1 weighted images were conducted with Echo time (TE) 1.7 s and repetition time (TR) 3.8 s. T2 weighted images were taken with Echo time (TE) 18.4 s and repetition time (TR) 53.6 s. Proton density (PD) images were done with Echo time (TE) 30 s and repetition time (TR) 1500 s.

On the other CBCT scans were performed using i-CAT Next generation (Imaging sciences International, Hatfield, PA, USA). Exposure factors were set at 120 kV, 37.07 mA and 26.9 s acquisition time. A 16 × 8 cm FOV was imaged using 0.2 mm voxel size. The patient position was standardized according to manufacturer’s instructions.

CBCT images were exported as digital imaging and communication in medicine (DICOM) files. They were then transferred to a third-party software (OnDemand 3DTM software, Cybermed Inc., Seoul, Korea) with a reconstruction interval set to 1.0 and 1.0 mm slice thickness. Two experienced oral and maxillofacial radiologists with 10 years of experience evaluated the CBCT and MRI images separately and disagreement was resolved by consensus.

### MRI image analysis

A maxillofacial and a medical radiologist with more than 10 years’ experience assessed the MRI scans together and reached a diagnosis by consensus. This diagnosis was considered the gold standard to which the CBCT findings were compared.

Each joint was assessed in both closed and opened mouth positions. Classification of the articular disc position was performed using sagittal oblique cuts by combining the criteria presented by Ahmad et al. [16] and Tasaki et al. [17] as follows:

*No disc displacement (NDD)*: in the corrected sagittal plane, in closed mouth position, in relation to the superior aspect of the condyle, the posterior band is located at 11:30–12:30 position, and the thin intermediate zone is found between the condyle and the articular eminence.

*Displacement of the disc*: in the corrected sagittal plane, in closed mouth position, in relation to the superior aspect of the condyle, the posterior band of the disc is located anterior to the 11:30 position, and the intermediate zone is located anterior to the condyle.

**Fig. 1:**
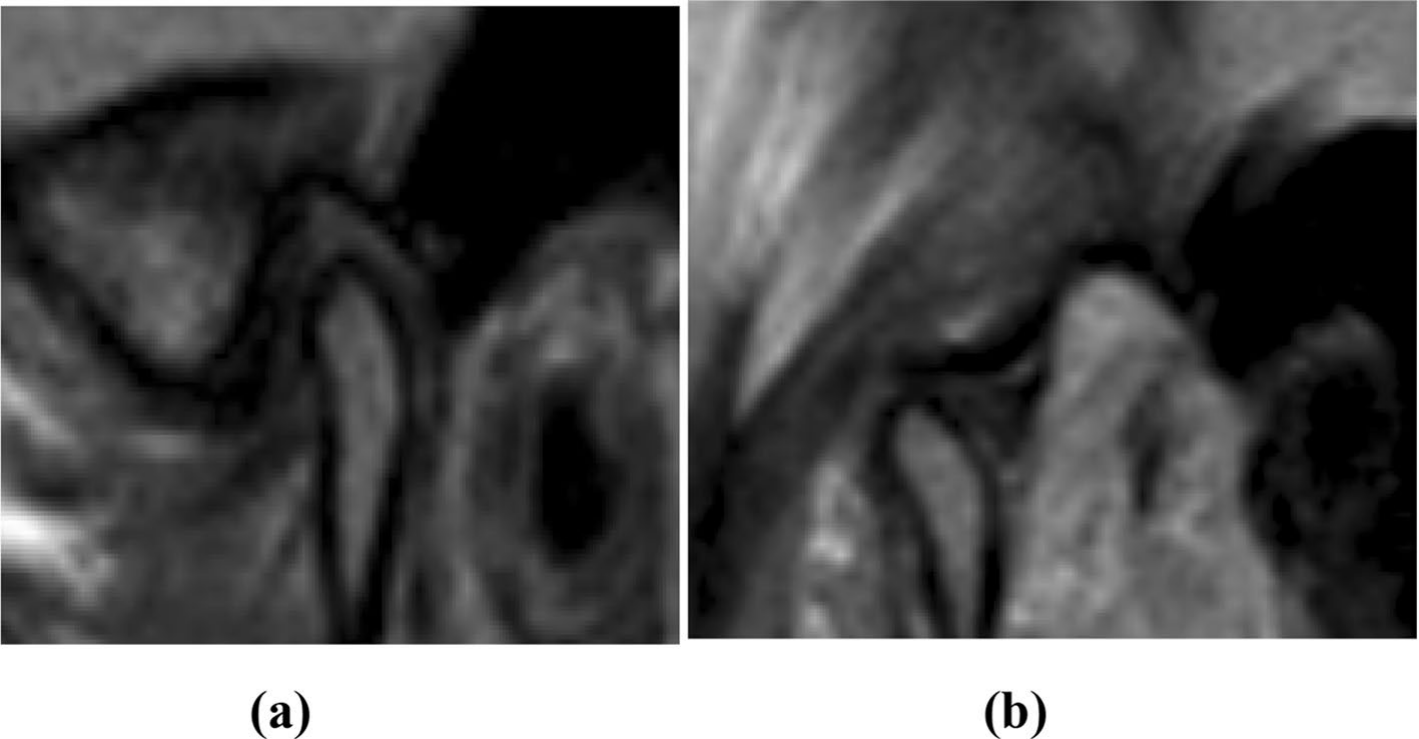
Sagittal proton density MRI images of the left TMJ. **a** Closed mouth position showing the posterior band of the disc is anterior to the 12:30 position in relation to the superior aspect of the condyle indicating anterior disc displacement. **b** Open mouth position showing the disc returning to the normal position suggesting ADDWR

*Anterior disc displacement with reduction (ADDWR)*: the displaced disc returns back to its normal position of 11:30–12:30 in relation to the condyle during the mouth-opening, and the intermediate zone is located between the condyle and the articular eminence (Fig. 1).

*Anterior disc displacement without reduction (ADDWOR)*: the displaced disc does not reduce to its normal superior position of 11:30–12:30 in relation to the condyle during the mouth opening movement, and the intermediate zone is located anterior to the condylar head.

*Posterior disc displacement with reduction (PDDWR)*: the disc is displaced posterior to the 12 clock position on top of the condyle in the closed position but resumes its normal position over the condyle while opening the mouth.

*Posterior disc displacement without reduction (PDDWOR)*: the displaced disc does not reduce to its normal position during mouth opening the disc remains turned at less than 11 clock with respect to the condyle in the open mouth position.

### CBCT image analysis

CBCT scans were assessed twice by two maxillofacial radiologists with more than 10 years of experience with one week interval in between to calculate the reliability of CBCT quantitative and qualitative assessment.

**Fig. 2:**
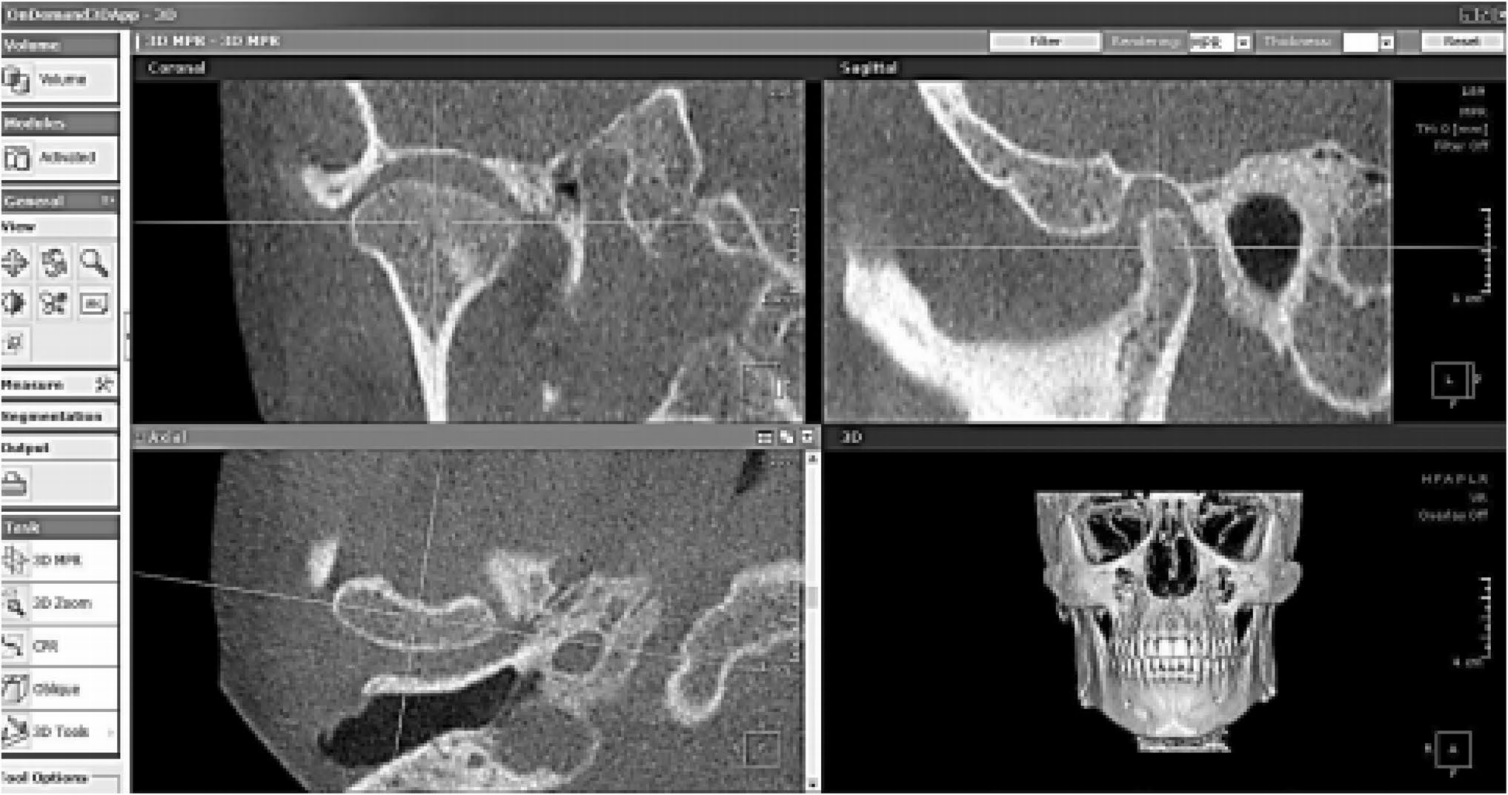
Standardized orientation of CBCT MPR views

On the multiplanar (MPR) screen; coronal, axial and sagittal views were reoriented to view the widest condyle dimension in each plane. The coronal plane was oriented on the axial window to pass through the condyle at its widest dimension mediolaterally. The sagittal plane was oriented on the axial window to be perpendicular to the coronal plane (Fig. 2).

**Fig. 3:**
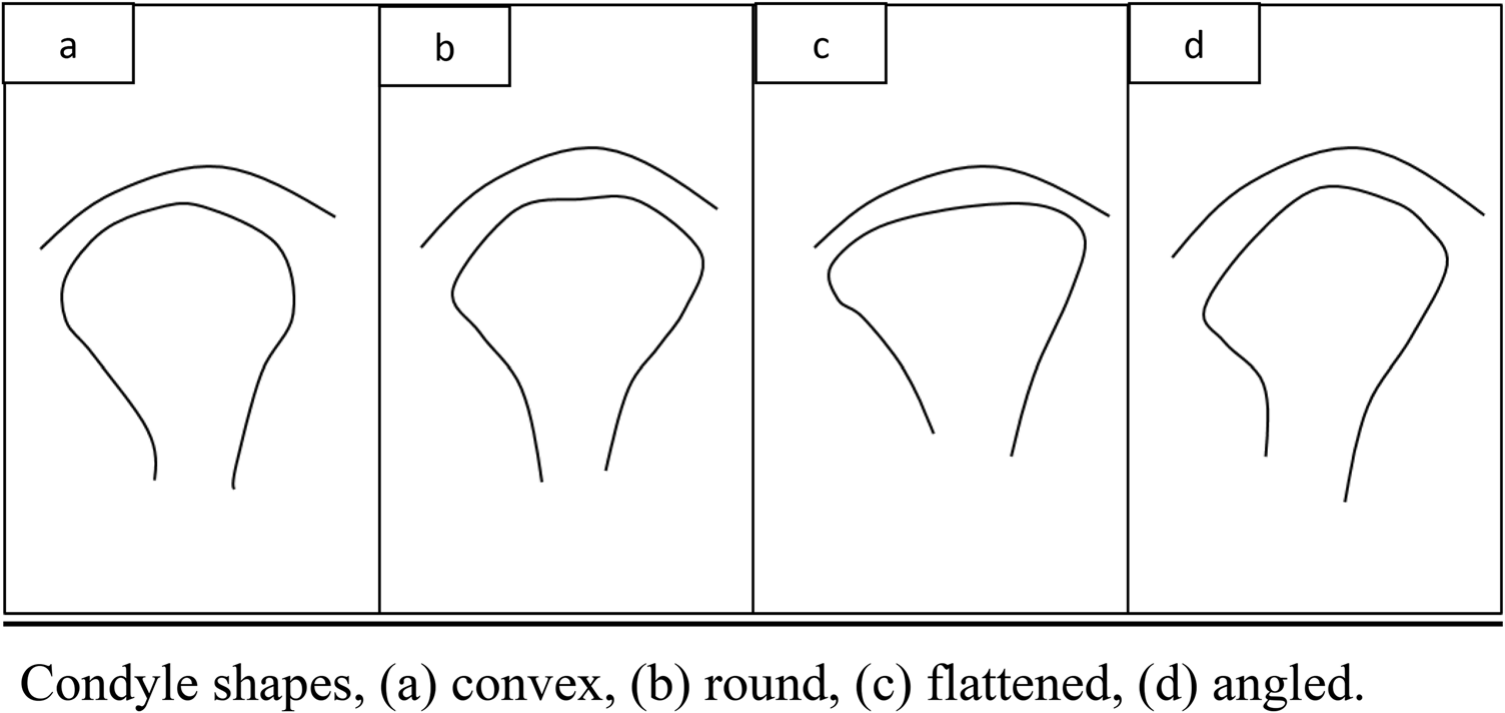
Condyle shapes, **a** Convex, **b** Round, **c** Flattened, **d** Angled

*Condylar shape*: condyle shape was assessed from the corrected coronal view, and it was classified into convex, flat, round or angled. Convex surface is identified when the surface is like a portion of an oval shape. Flat surface shows a nearly straight surface between the right and left summits. Round surface is selected when the upper surface resembles half a circle. Angled surface has a sharp bend on the upper surface of the condyle as shown in (Fig. 3).

**Fig. 4:**
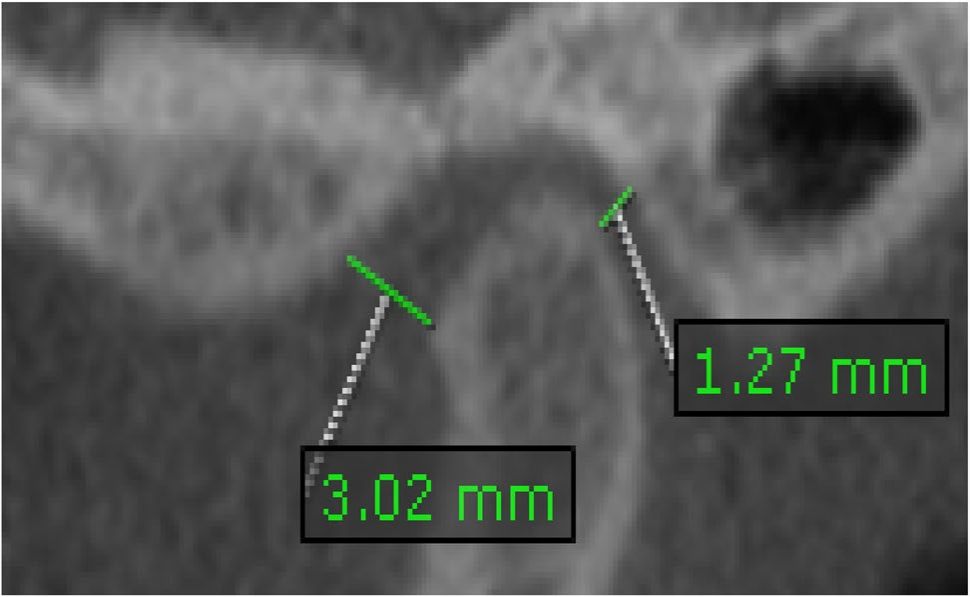
CBCT corrected sagittal view of the condyle showing measurement of the anterior (3.02 mm) and posterior joint space (1.27 mm) to determine the condyle position

*Condylar position*: condylar position was evaluated from the corrected sagittal view. To assess the condylar position, the anterior and posterior joint spaces were measured. A line perpendicular to the point A (A: most prominent anterior point of the condyle) was extended to the opposing posterior eminence slope. The distance between the point A and the posterior eminence slope is the measure of anterior joint space (AS). Another line perpendicular to the point P (P: most prominent posterior point of the condyle) was extended to the opposing bone wall of the joint. The distance between the point P and the opposing bone on that line is the posterior joint space (PS) as shown on (Fig. 4). AS = PS indicates concentric condyle position, AS > PS indicates posterior condylar position, while AS < PS indicates anterior condylar position [18].

**Fig. 5:**
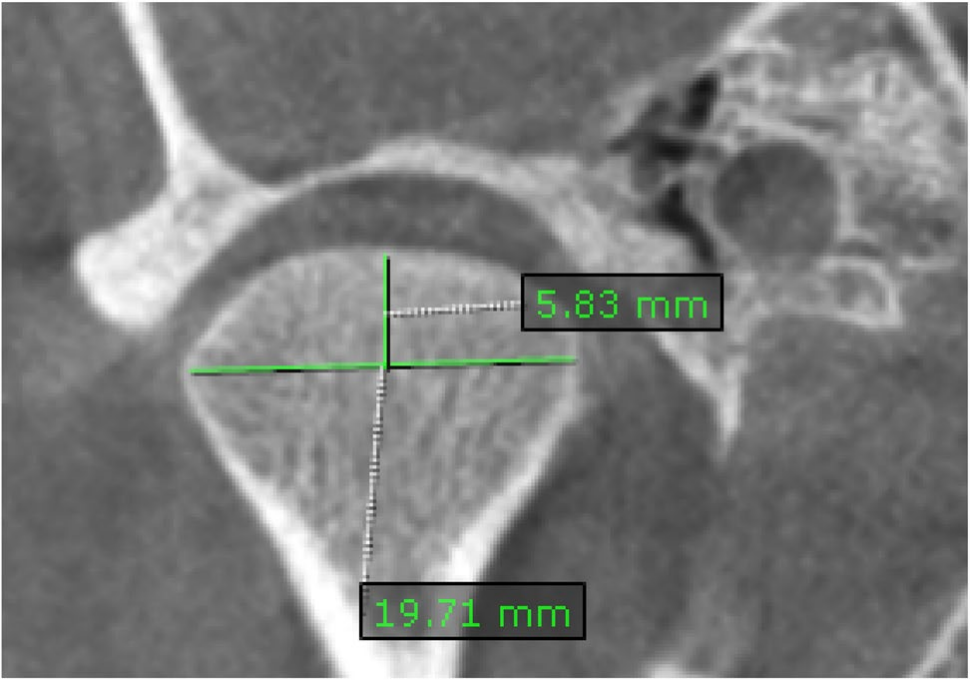
Corrected coronal view showing the mediolateral condyle width along the axial plane passing through the condyle and the condyle height along the sagittal plane as the perpendicular distance from the top of the condyle till the axial plane

*Condylar height and condylar width*: condylar height and mediolateral width measurements were performed on the corrected coronal view with the largest condyle dimensions as shown on (Fig. 5). The condyle width was measured between the most prominent points on the right and left slopes of the head of the condyle. At the center of the width, a perpendicular line was extended to the roof of the condyle. The length of this line represents condylar height [9].

**Fig. 6:**
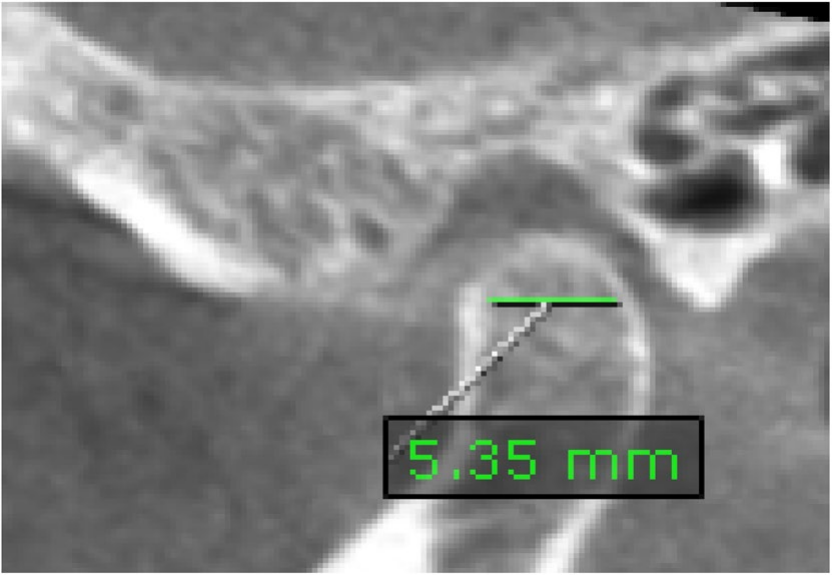
Corrected sagittal view showing the anteroposterior condyle dimension

*Condylar depth*: it is the antero-posterior dimension of the condyle. It was measured on the corrected sagittal view on a line between the most prominent anterior (A) and posterior (P) points of the condylar head [19]. (Fig. 6).

Seventeen patients (34 TMJs) were included in the present study having a Helkimo index score of 5–20. MRI imaging revealed that 20 TMJs showed ADDWR, 7 TMJs showed normal disc position, 6 TMJs had ADDWOR and 1 TMJ showed posterior disc position. TMJs showing ADDWOR and posterior disc position were excluded. So, we ended up comparing 20 TMJs having ADDWR with 7 TMJs showing normal disc position.

### Statistical analysis

All data were collected and tabulated. The data were analyzed by SPSS (version 20), while Microsoft office Excel was used for data handling and graphical presentation. Quantitative variables were described by the mean, standard deviation (SD), range (minimum–maximum), standard error (SE) and 95% confidence interval of the mean. Qualitative categorical variables were described as frequencies and percentages.

Shapiro–Wilk test was used to test normality of all quantitative variables for further choice of appropriate parametric or non-parametric tests. Independent samples *t*-test was applied to compare the means of the two groups as almost all variables were found to be normally distributed.

For nominal variables, chi-squared test of independence and fisher exact test were applied, and the correlation was assessed using Cramer’s V and Contingency Coefficient measures.

Logistic regression model was established to predict the probability of anterior disc displacement with reduction depending on condyle height, width and depth.

Significance level was set at *P* ≤ 0.05 (S); while *P* ≤ 0.01 was considered highly significant (HS). Two tailed tests were assumed throughout the analysis for all statistical tests.

## Results

A total of 17 patients were included in our study (5 males and 12 females) with age range (18–55) and mean age 33.3 ± 16.23 years. Of the 34 total TMJs studied, seven showed NDD, 20 presented with ADDWR, six showed ADDWOR and one condyle was posteriorly displaced without reduction. Only two out of the seven NDD condyles showed condylar changes. The case with PDD showed no condylar changes. However, all the ADDWOR TMJs showed osteoarthritic changes. TMJs having ADDWOR or posterior displacement were excluded from the study. Then we compared TMJs showing ADDWR with NDD.

Regarding the correlation between ADDWR and condylar shape, the correlation was of moderate strength and statistically significant. Angled shaped condyle is the dominant shape in cases of ADDWR as shown in Tables 2 and 3.

**Table 2:** Chi-square and Fisher exact test showing condylar shape in TMJs with anterior disc displacement with reduction vs normal TMJs

Disc position		Condyle shape		Total	Pearson chi-Square	*P* value	Fisher exact test probability
		Angled	Convex				
Normal	2		5	7	6.17	0.01298*	0.02345
	28.6%		71.4%	100.0%			
Anterior disc displacement with reduction	16		4	20			
	80.0%		20.0%	100.0%			
Tota**l**		18	9	27			
		66.7%	33.3%	100.0%			

^**^Highly significant at *P* ≤ 0.01

*Significant at *P* ≤ 0.05

**Table 3:** Symmetric measures of correlation between anterior disc displacement with reduction and condyle shape

Nominal by nominal	Value	*P* value
Phi	0.478	0.01298*
Cramer's V	0.478	0.01298*
Contingency coefficient	0.431	0.01298*

^**^Highly significant at *P* ≤ 0.01

*Significant at *P* ≤ 0.05

The correlation between ADDWR and condylar position in the glenoid fossa was of moderate strength and statistically highly significant. Posterior condylar position is the main position in cases of ADDWR as shown in Tables 4 and 5.

**Table 4:** Chi-square and Fisher exact test showing the differences between normal disc position and anterior disc displacement with reduction regarding condylar position in the glenoid fossa

Disc position	Condylar position in glenoid fossa		Total	Pearson chi-square	*P* value	Fisher exact test probability
	Centric	Posterior				
Normal	5	2	7	7.92	0.00489**	0.01139
	71.4%	28.6%	100.0%			
Anterior disc displacement with reduction	3	17	20			
	15.0%	85.0%	100.0%			
Total	8	19	27			
	29.6%	70.4%	100.0%			

^**^Highly significant at *P *≤ 0.01

*Significant at *P* ≤ 0.05

**Table 5:** Symmetric measures of correlation between anterior disc displacement with reduction and condylar position in the glenoid fossa

Nominal by nominal	Value	*P* value
Phi	0.542	0.00489**
Cramer's V	0.542	0.00489**
Contingency coefficient	0.476	0.00489**

^**^Highly significant at *P* ≤ 0.01

*Significant at *P* ≤ 0.05

Condylar width, height and depth were smaller in patients with ADDWR compared to those with NDD as shown in Table 6.

**Table 6:** Independent samples *t*-test showing the differences between normal disc position and anterior disc displacement with reduction regarding condylar height, width and depth

Disc position		Condyle height	Condyle width	Condyle depth
Normal	*N*	7		
ADDWR		20		
Normal	Mean	4.65	18.83	4.72
ADDWR		3.55	16.36	3.76
Normal	SD	0.87	1.58	1.31
ADDWR		0.66	1.99	0.98
Normal	SEM	0.33	0.60	0.49
ADDWR		0.15	0.45	0.22
Mean difference		1.10	2.47	0.96
SE difference		0.32	0.84	0.47
95% Confidence interval of the difference	Lower	0.45	0.75	0.00
	Upper	1.75	4.20	1.92
*T*		3.49	2.96	2.06
df		25	25	25
*P* value		0.00182**	0.00663**	0.05038*

^**^Highly significant at *P* ≤ 0.01

*Significant at *P* ≤ 0.05

Binary logistic regression analysis was formulated to predict ADDWR from CBCT condylar dimensions as follows:

$$P\, = \,\exp \, (23.45 - 0.92 W - 2.32H\, + \,0.85D)/(1 + \,\exp \, (23.45 - 0.92 W - 2.32H\, + \,0.85D))P\, = \,\exp \, (23.45 - 0.92 W - 2.32H\, + \,0.85D)/(1 + \,\exp \, (23.45 - 0.92 W - 2.32H\, + \,0.85D))P\, = \,\exp \, (23.45 - 0.92 W - 2.32H\, + \,0.85D)/(1 + \,\exp \, (23.45 - 0.92 W - 2.32H\, + \,0.85D))$$

*P*: the probability of ADDWR is one (or in other words the probability of normal disc position is zero), *W*: width in mm, *H*: height in mm, *D*: depth in mm.

Substituting the values of *W*, *H* and *D* gives the probability of the disc position being ADDWR.

## Discussion

In this study we examined the ability of CBCT assessment of the osseous components of the TMJ to predict ADDWR. The final objective is to predict all types of internal derangement from CBCT images of the TMJ. However, ADDWOR and PDD were excluded from the study. This is because regression analysis can forecast the value of one dependent variable (ADDWR present vs absent) from the values of one or more independent variables (condylar width, depth, height). Therefore, a different regression analysis is needed to predict ADDWOR or PDD. The number of TMJs having ADDWOR or PDD was not enough to provide statistical validity for this type of analysis. More patients are being recruited to further study the other forms of internal derangement.

Our results showed a significant correlation between ADDWR and both condylar shape and position. Moreover, all linear condylar dimensions were significantly reduced in ADDWR compared to normal joints.

Condylar shape is an indicator of joint health and function [20]. Normal variation in the morphology of the condyle occurs with age, gender, facial type, functional load and malocclusion [19]. In our study, there was a significant correlation between ADDWR and condyle shape. The angled shape condyle was the prominent shape in ADDWR (80%), while the convex shape condyle was the dominant shape in normal condyles (71.4%). The predominance of the angled shape condyle in ADDWR may be attributed to changes in the amount and distribution of stresses that the condyle is subjected to which alter the normal convex form into an angled shape. Similarly, Durgha et al. [21] stated that the normal condylar head has a convex configuration throughout. Sülün et al. [6] found a higher prevalence of ADDWR in angled condyles. Opposingly, De Farias et al. [22] found that there is no correlation between the morphology of the condyle and TMJ disc displacement.

Regarding condylar position, concentric condylar position was predominant in normal disc position while posterior condylar position was predominant in ADDWR (85%). Similarly, Gateno et al. [23] stated that the head of the condyle in patients with anterior disc displacement was located more posterior in the mandibular fossa when compared with the normal group. Moreover, Cho and Jung [24] stated the centric position of condyle was more common in the asymptomatic group while the posterior position of the condyle was more prevalent in the symptomatic group. Furthermore, Dumas et al. [7] found that symptomatic condyles generally showed a more posterior position. Rabelo et al. [8] reported a correlation between anterior disc displacement and an increased size of anterior joint space. On lateral tomograms, Almasan et al. [15] observed that anterior joint space was significantly larger in cases with ADDWR compared to the group with NDD. On the contrary, Lelis et al. [23] and Katzberg et al. [25] did not find a significant difference between joints diagnosed with ADDWR and those with normal disc position regarding the condylar position in the fossa.

Concerning condylar width, in the present study the condylar width was smaller in patients with ADDWR than those with NDD. That decrease in condylar width may be caused by the resorption of the lateral pole of the condyle in the early stages of TMD. Choukas and Sicher [26] explained that the TMJ disc is firmly attached to the medial and lateral poles of the posterior aspect of the condyle. When the disc is displaced further anteriorly, it stretches the medial and lateral attachments causing resorption to the related side of the condyle. The postero-superior surface of the lateral pole of the condyle is especially prone to resorption [27]. Likewise, Okur et al. [28] measured condyle width using CT, and reported significant differences between NDD and ADDWR. Seo et al. [9] found that condylar width is less in ADDWR compared to NDD. On the contrary, Imanimoghaddam et al. [29] found that there is no significant correlation between ADDWR and changes in condylar width.

Regarding condylar height, in the current study the correlation between disc position and condylar height was highly significant. Condylar height was smaller in condyles with ADDWR compared to those NDD. These results oppose those published by Seo et al. [9] who found that there was no significant difference in condylar height between NDD and ADDWR.

Regarding condyle depth, in our study condylar depth was significantly smaller in condyles with ADDWR compared to condyles having NDD. Similarly, Yasa et al. [30] observed that the condylar depth was smaller in patients with ADDWR compared to those with NDD. Opposingly, Seo et al. [9] and Imanimoghaddam et al. [29] found that there was no significant difference in condylar depth between NDD and ADDWR.

Many studies have attempted to clarify the changes in condylar morphology, position and dimensions associated with ADDWR. The morphologic changes are caused by the regressive changes in the condyles associated with TMJ disc displacement [31]. Osteoarthritis begins in the condylar cartilage. Condylar resorption is thought to be caused by normal stress on aberrant cartilage [32] which leads to changes in condylar dimensions in patients suffering from ADDWR.

Chiappe et al. [33] used logistic regression to predict ADDWR using 12 occlusal variables: partial unilateral posterior cross-bite, anterior open occlusal relationship, vertical anterior overlap, complete unilateral posterior cross-bite, anterior horizontal overlap (overjet; normal value bilateral first angle class canine and first molar relationship plus dynamic occlusion features such as length and symmetry of retruded contact position [RCP] to maximum intercuspation [MI] slides. This publication revealed only a weak association between three occlusal features (RCP ⁄MI slide, mediotrusive, absence of canine guide in lateral movements) and ADDWR.

Manfredini et al. [34] used logistic regression to predict TMJ clicking from features of dental malocclusion. The subjects were divided into two groups a TMJ clicking and a no-TMJ clicking group. Seven occlusal features were recorded for each patient: (1) posterior crossbite, (2) overbite, (3) open bite, (4) overjet, (5) mediotrusive and (6) laterotrusive interferences and (7) retruded contact position to maximum intercuspation slide length. They concluded that the contribution of malocclusion features in predicting TMJ clicking is minimal.

In this study, condylar dimensions (height, width and depth) were put into the model of binary logistic regression to find a cut-off point for condylar dimensions below which ADDWR is suspected.

A positive result of the logistic regression equation, together with angled shaped, posteriorly positioned condyle point out to ADDWR.

## Conclusion

Our results showed a significant correlation between condylar shape, position and dimensions and ADDWR of the TMJ. Logistic regression could be used to predict the probability of anterior disc displacement with reduction by substituting the values of condylar height, width and depth in the formula.

## Recommendations

Further large-scale studies are needed to test the accuracy of this logistic regression in accurately predicting ADDWR. Achieving computerized condylar measurements and automated prediction of internal derangement from CBCT image analysis software would be an important milestone.
